# Diffuse Pleural Mesothelioma: A Challenge in Early Diagnosis

**DOI:** 10.7759/cureus.74998

**Published:** 2024-12-02

**Authors:** Ancuta-Alina Constantin, Florin Dumitru Mihaltan, Angela-Stefania Marghescu, Gabriela Andreea Craciunica

**Affiliations:** 1 Pulmonology, Institute of Pneumology “Marius Nasta”, Bucharest, ROU; 2 Respiratory Medicine, University of Medicine and Pharmacy “Carol Davila”, Bucharest, ROU; 3 Histopathology, Institute of Pneumology “Marius Nasta”, Bucharest, ROU; 4 Internal Medicine, Clinical Hospital "Prof. Dr. Theodor Burghele", Bucharest, ROU

**Keywords:** adjuvant polychemotherapy, biopsy, malignant pleural mesothelioma, pleural effusion, pleurectomy

## Abstract

We present the clinical case of a 58-year-old female patient, a smoker with occupational exposure to respiratory toxins, who was admitted to our clinic following evaluation in an emergency department, where she was diagnosed with a moderate right pleural effusion. Upon admission, the patient exhibited respiratory symptoms, including progressive dyspnea with a moderate exertion threshold, right posterior pleuritic chest pain radiating anteriorly, occasional episodes of low-grade fever, and persistent febrile symptoms lasting approximately two weeks. In this clinical context, the diagnostic process was guided by the presence of right pleural effusion syndrome, which was refractory to conservative medical therapy. This necessitated a careful and stepwise expansion of investigations, ultimately leading to the diagnosis of malignant pleural mesothelioma. This case underscores the diagnostic challenges posed by pleural effusion, the necessity of adhering to the diagnostic algorithm, and the critical role of the multidisciplinary team. The diagnostic approach, often complex and challenging, necessitates a multidimensional strategy that integrates the correlation and synthesis of data obtained through anamnesis, alongside advanced diagnostic procedures such as pleural biopsy, which remains the gold standard. This comprehensive process is essential for formulating a diagnostic suspicion, with the final diagnosis intended to be one of exclusion.

## Introduction

Malignant pleural mesothelioma (MPM) is an uncommon but highly aggressive malignancy that arises in the pleural lining of the lungs, primarily due to inhaled asbestos fibers. These microscopic fibers embed in the pleura, initiating cellular inflammation and genetic damage that, over many years, often 20 to 50, can progress to mesothelioma. MPM is notoriously challenging to diagnose early, as initial symptoms like chest pain, dyspnea, and pleural effusions are often nonspecific and mimic other pulmonary conditions [1].

The disease is generally staged I through IV, with higher stages indicating greater spread to lymph nodes, nearby structures, or distant sites, which complicates treatment. Standard therapies include surgery, such as pleurectomy/decortication or extrapleural pneumonectomy, combined with chemotherapy (often pemetrexed and cisplatin) and radiation therapy. Recent advancements in immunotherapy, particularly checkpoint inhibitors, are providing new hope, albeit limited, for extending survival. Despite ongoing research, the prognosis for MPM remains poor, making early diagnosis and experimental therapies critical areas of focus in mesothelioma management [2].

## Case presentation

A 58-year-old female patient was admitted to our clinic for specialized investigations and appropriate therapeutic management following the diagnosis of a right pleural effusion, indicated at an emergency medical service where she initially presented. She was a smoker with a 15-pack-year history and 15 years of occupational exposure to respiratory toxins from working in the tobacco industry, multiple comorbidities, including grand mal epilepsy, stage II hypertension, hypertensive heart disease with preserved ejection fraction, grade I obesity, a hysterectomy in 2001 for uteroplacental apoplexy, and surgically induced menopause at age 34.

**Figure 1:**
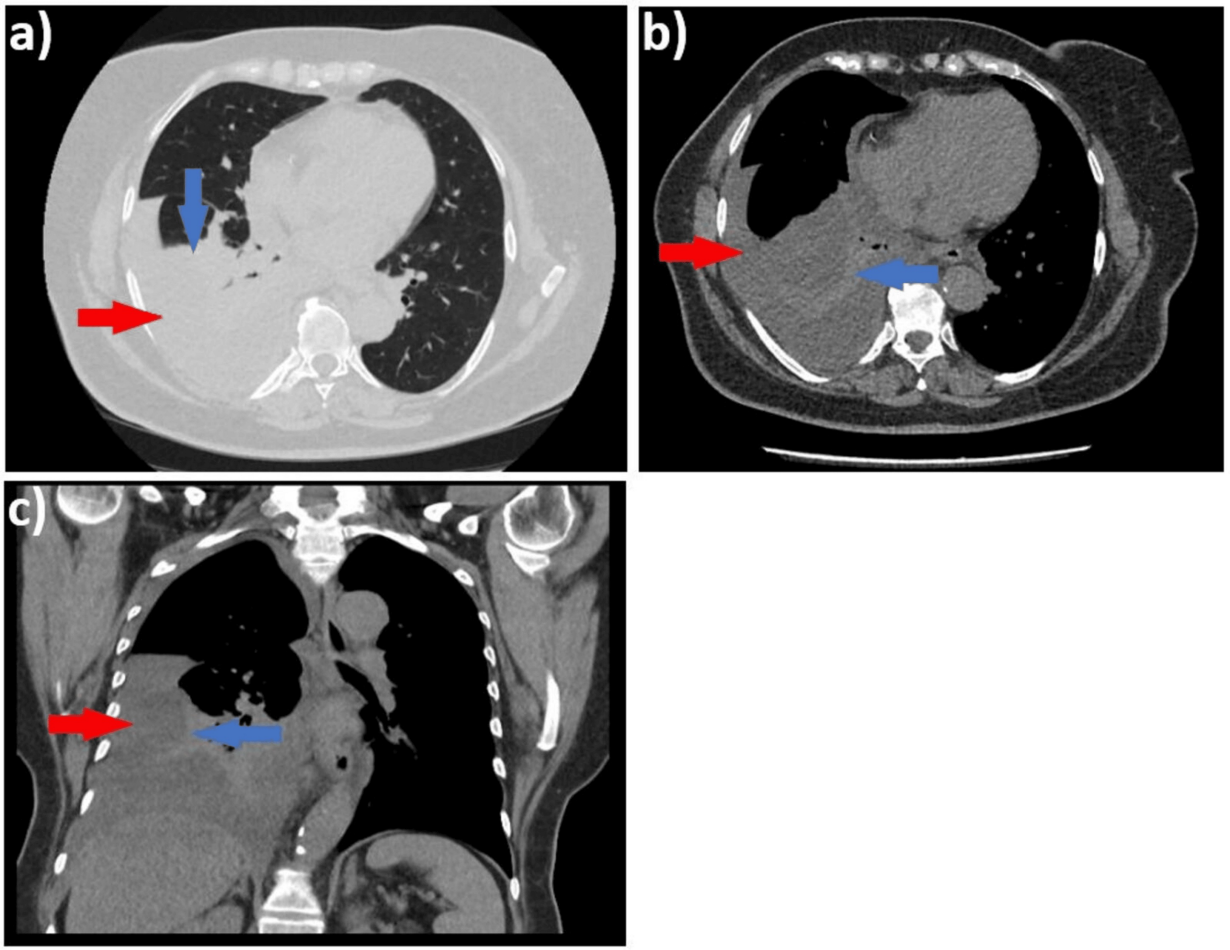
Non-contrast chest CT scan, (a) lung window, axial plane; (b) mediastinal window, axial plane; (c) coronal plane, mediastinal window, showing pleural effusion (red arrows) and collapsed lung (blue arrows)

The patient reported an insidious onset of symptoms, including progressive dyspnea with moderate exertion (mMRC score 2), right posterior chest pain radiating to the anterior chest, and intermittent episodes of low-grade fever (37.2°C to 38°C). Given her poor general condition and these symptoms, she presented to the emergency department, where a chest X-ray was performed, and antibiotic and symptomatic treatment was also initiated. The X-ray (not provided) revealed changes suggestive of a moderate right pleural effusion. As the COVID-19 pandemic had reached Romania at this time (January 2021), patients' hesitation to seek medical care in hospitals was quite significant, with the major recommendation from authorities being self-isolation and social distancing. It is likely that the delay in diagnosis coincided with the postponement of hospital visits in the pandemic context. Further investigation with a chest CT scan confirmed the presence of a significant right pleural effusion, fused at the level of the oblique fissure (Figure 1, red arrows), causing passive collapse of the subpleural lung parenchyma (Figures 1, blue arrows).

The patient was then referred to our clinic and directed to the local pneumology service. Considering the persistent symptoms and right pleural effusion, a right thoracentesis was performed for diagnostic and therapeutic purposes, draining approximately 600 ml of serous fluid with biochemical characteristics of exudate and cytology showing atypical cells suggestive of malignancy. This required a right pleural biopsy, which, however, was non-diagnostic due to the chronic inflammatory changes described.

Bioumoral, at the time of admission, we observed a non-specific biological inflammatory syndrome with an erythrocyte sedimentation rate (ESR) greater than twice the upper limit of normal, and subsequently developed mild thrombocytosis (platelets (10^3^/uL) = 451,000/mm³) (Table 1).

**Table 1:** Laboratory blood test AST: aspartate transferase; ALT: alanine transaminase; ESR: erythrocyte

Laboratory test	Patient values at admission	Normal range
Hemoglobin (g/dL)	12.70	11.7-18
Hematocrit (%)	38.90	36.6-44
Total leukocyte count (10^3^/µL)	8.01	4.49-12.68
Lymphocyte %()	31.70	18.3-45.7
Monocyte (%)	4.9	4.2-11.8
Eosinophils (%)	3	0.2-5.3
Basophil (%)	0.90	0.1-1
Platelets (10^3^/µL)	451.000	173-390
Creatinine (mg/dL)	0.39	0.51-0.95
Urea (mg/dL)	23	17-43
AST (U/L)	18	0-35
ALT (U/L)	25	0-35
ESR (mm/h)	60	2-30
Fibrinogen (mg/dL)	429	238-498

Complementary to the chest CT examination, a fiberoptic bronchoscopy with bronchoalveolar lavage was performed. The findings revealed a normal larynx, diffuse bronchitis, and no visible proliferative elements in the examined areas. Microbiological tests for non-specific flora, mycology, and Ziehl-Neelsen staining showed no evidence of pathogenic organisms. During the diagnostic process, we considered thoracoscopy with pleural biopsy to be a mandatory step, but the patient decided to postpone the investigation, opting instead for a watchful waiting approach and dynamic imaging follow-up.

**Figure 2:**
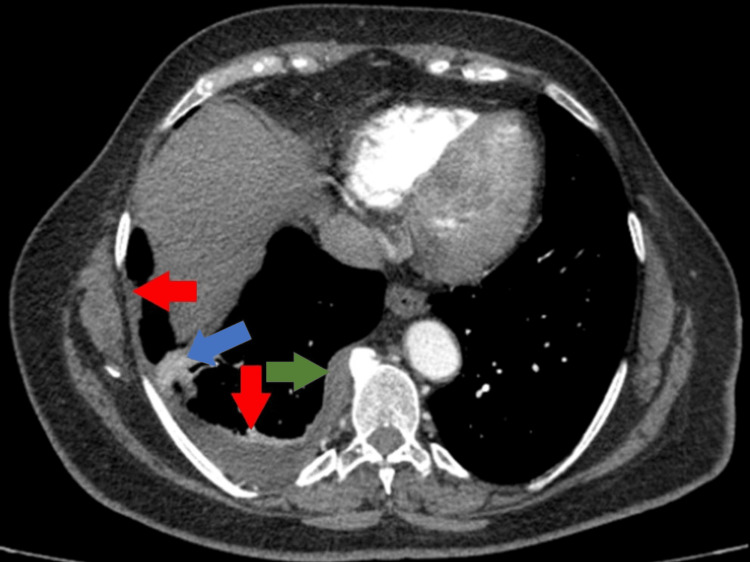
Contrast chest CT scan (mediastinal window, axial plane) showing recurred pleural effusion, pleural fluid collections, subpleural lamellar pulmonary consolidation and pleural thickening

The three-month follow-up (March 2021) chest CT revealed lesion polymorphism, characterized by a moderate right pleural effusion that recurred after drainage. This was associated with encysted focal pleural fluid collections in the posterior-basal region, approximately 16 mm thick (Figure 2, red arrow), contrast enhancement subpleural lamellar pulmonary consolidation (Figure 2, blue arrow), focal pleural thickening in the paravertebral and posterior regions on the right side, measuring 6 mm (Figure 2, green arrow), a calcified subpleural micronodule in the lateral thoracic area of the left upper lobe, and left supraclavicular lymphadenopathy measuring 15 x 11 mm.

The recurrent nature of the pleural effusion, previously suspicious cytological changes, and the persistence of the clinical picture refractory to prolonged antibiotic therapy (approximately one month) and associated analgesic medication, with ongoing episodes of low-grade fever, worsening dyspnea, and right lateral-posterior chest pain-were key factors in the decision to proceed with thoracoscopy with pleural biopsy. Under general anesthesia with selective orotracheal intubation, a right thoracoscopy with pleural biopsy was performed.

An iterative right lateral incision was made, providing pleural access at the level of the fifth intercostal space. Upon entering the pleural cavity, the parietal pleura was found to be thickened, hard, whitish, and friable, with multiple generalized pleuro-pulmonary adhesions and several encysted liquid collections containing serous citrine fluid. Multiple pleural fragments were collected and sent for histopathological examination, after which approximately 400 ml of serous citrine fluid was evacuated.

The histopathological examination of the pleural biopsy revealed the specimen was entirely included and examined in two serial sections. The sample consisted of fibrin masses and multiple fragments with pleural connective structure, fibro-collagenous, within which, in certain areas, a carcinomatous tumoral infiltration with glandular arrangement was identified, leading to the conclusion of pleural metastasis of adenocarcinoma aspect, with an unspecified primary origin.

The immunohistochemical tests performed following the histopathological examination reconsidered the entire diagnostic context, supporting the appearance of diffuse malignant pleural mesothelioma of the epithelioid subtype. The discrepancy between the two results raised additional concern, leading to the reevaluation of the samples in a tertiary laboratory, which confirmed the diagnosis of epithelioid mesothelioma with a tubular pattern.

During this crucial period dedicated to the diagnostic process, the patient’s symptoms escalated, with the clinical picture worsening by increased right lateral chest pain, a weight loss of 15 kg over the past two months, profuse night sweats, and malaise.

Furthermore, the case was discussed by a multidisciplinary team consisting of a pulmonologist, oncologist, and thoracic surgeon. Considering the absence of secondary hepatic, adrenal, and contralateral pulmonary determinations, as revealed by the investigations conducted privately, the consensus was to implement a comprehensive therapeutic plan, which includes a quasi-total right pleurectomy and adjuvant chemotherapy.

**Figure 3:**
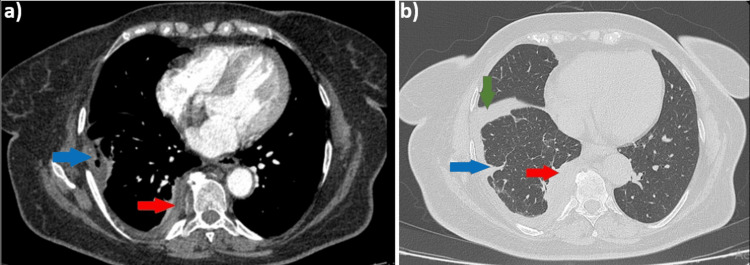
Contrast Chest CT scan, (a) mediastinal window, axial plane; (b) lung window, axial plane, describing paravertebral pleural thickening, pleural effusion, and fluid accumulation in the right fissure.

The preoperative imaging reevaluation revealed the progression of pleural thickening in the right paravertebral region, and mild contrast enhancement, measuring approximately 17 mm in thickness (Figure 3, red arrows). Additionally, a small right pleural effusion with encysted areas, up to 15 mm thick, in the subpleural region was noted (Figure 3, blue arrows), along with fluid accumulation in the right fissure (Figure 3b, green arrow).

**Figure 4:**
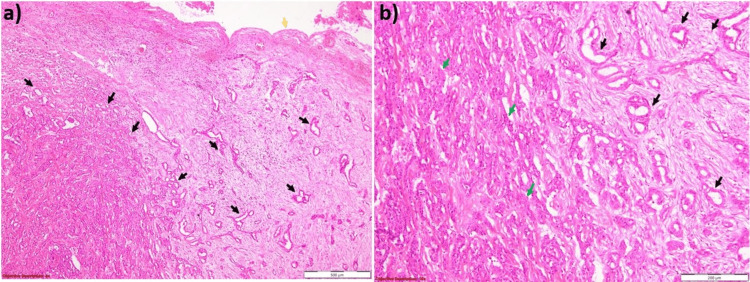
Histopathology samples indicating epithelioid mesothelioma (a) Epithelioid mesothelioma (black arrows) located in the visceral pleura (orange arrows), displaying tubular, trabecular, and solid architectural patterns, along with prominent stromal desmoplasia (H&E, 40x); (b) Epithelioid mesothelioma composed of relatively monotonous neoplastic cells with prominent nucleoli, thickened nuclear membranes, and eosinophilic cytoplasm, arranged in tubular (black arrows) and trabecular (green arrows) structures (H&E, 100x).

The extensive surgical intervention involved quasi-total pleurectomy, resection of a pericardial fragment, and resection of a diaphragmatic fragment. The procedure was extremely bleeding and difficult, but it had a favorable postoperative outcome. The histopathological reevaluation of the excised biopsy material confirmed the diagnosis of epithelioid mesothelioma (Figure 4).

Integration into the Oncology Department with the initiation of adjuvant polychemotherapy in six cycles with cisplatin-pemetrexed was the complementary stage to the surgical treatment. Shortly thereafter, the patient was diagnosed with COVID-19, presenting with a moderate clinical form, which slowed the therapeutic efforts for the neoplastic disease. The patient was regularly evaluated as part of oncological monitoring, with maintenance therapy using pemetrexed until January 2023, and her evolution remained favorable. The first postoperative CT scan reevaluation at six months noted minimal residual post-operative pulmonary changes on the right lung, no detectable pleural thickening on CT, and no signs of locoregional tumor recurrence. Subsequently, imaging assessments performed every three to six months over the next 33 months postoperatively continued to show favorable results, with no signs of local recurrence or regional extension.

**Figure 5:**
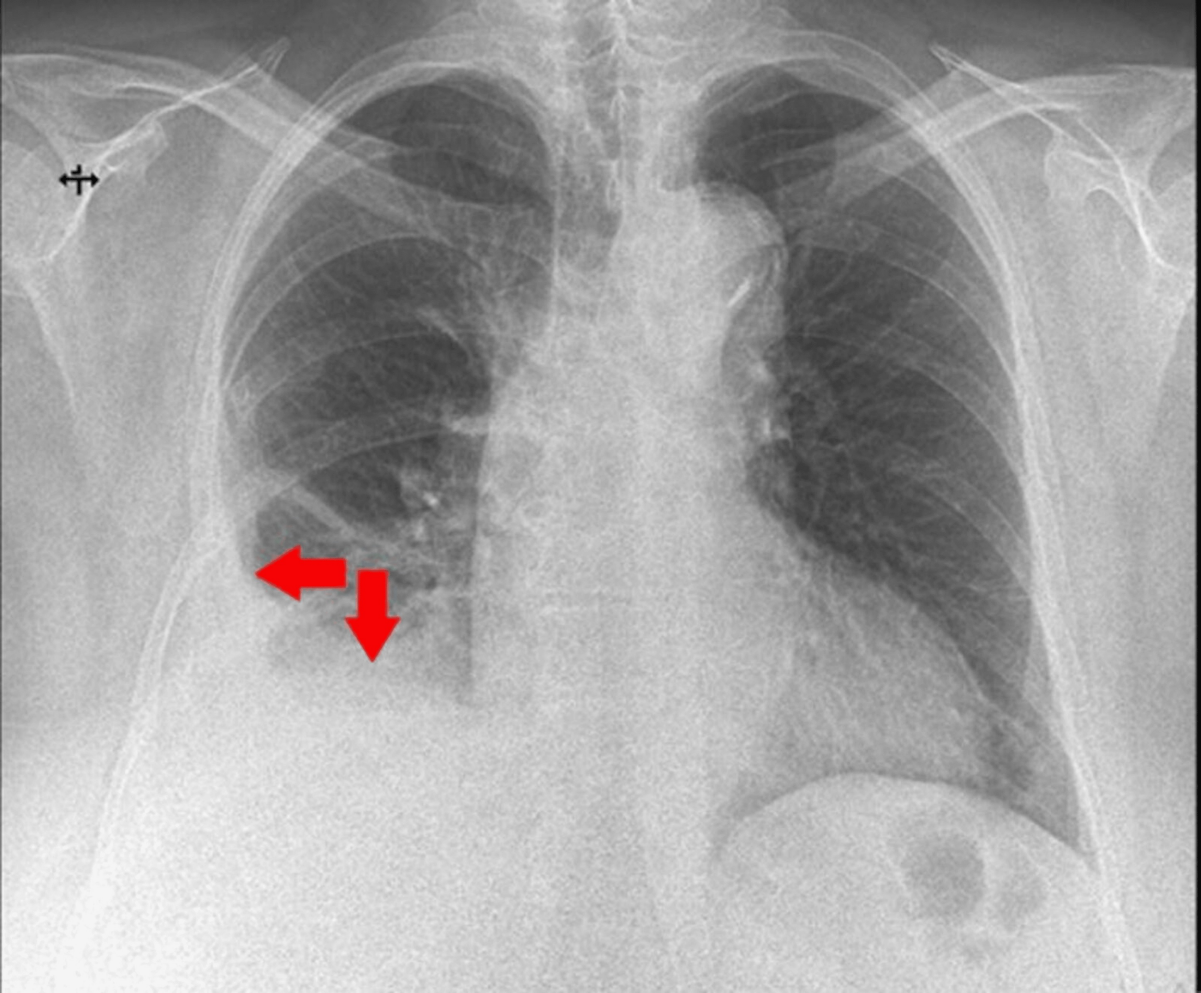
Chest X-ray (posteroanterior view), showing a small to moderate right pleural effusion

The patient's condition became unstable and difficult in December 2023, with the reappearance of the initial symptoms. Radiological imaging confirmed the recurrence of a small to moderate right pleural effusion (Figure 5). The suspicion of disease recurrence was raised, and it was confirmed through CT imaging.

**Figure 6:**
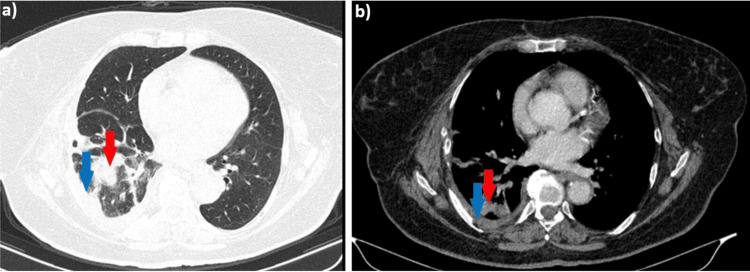
. Contrast chest CT scan, (a) lung window, axial plane; (b) mediastinal window, axial plane, showing a nodular pulmonary lesion and pleural effusion

Later, in May 2024, nodular pulmonary lesion was noted, mild contrast enhancement, newly appeared in the lower third of the right pulmonary field (Figure 6, red arrows). As the lesion increased in size, with a minimal recurrence of the pleural effusion on the CT scan (Figure 6, blue arrows), it was decided in June 2024 to initiate stereotactic external radiotherapy up to a total dose of 50 Gy, which was well-tolerated by the patient.

After irradiation, approximately two weeks later, the patient tested positive for COVID-19 again, developing a relatively mild infection that did not require extensive therapeutic management.

## Discussion

MPM is a primitive neoplasm of the pleura, universally fatal, with an average reported survival rate between 9 and 18 months from the time of diagnosis. Moreover, statistics show that less than 5% of patients survive beyond five years [1,3]. A direct causal relationship with asbestos exposure has been proven, making asbestos the most significant risk factor associated with the subsequent development of MPM [2]. However, only a small percentage of those exposed to asbestos develop the disease. A dose-response relationship and a long latency period between the initial exposure and the onset of the disease are noted, estimated at approximately 20-40 years [1,4,5].

Distinctively, and to a lesser extent, other factors implicated in the etiology of the disease, besides asbestos, include erionite, therapeutic radiation, genetic mutations, and Simian Virus 40 (SV40). Studies have shown that in families with malignant pleural mesothelioma, an increased risk of the disease correlates with the number of diagnosed descendants. This observation seems to be based on the genetic component that increases the risk of neoplasia in these families, specifically mutations in the *BRCA-1* and *BAP* genes, and tumoral suppressor genes involved in DNA transcription. A significant proportion of patients present mutations in genes that cause malignant mesothelioma, particularly those that lead to peritoneal mesothelioma [1,6,7].

Regarding epidemiological data, there are no exact estimates of the prevalence of the disease, as figures vary greatly depending on the geographical area, and the incidence and mortality of MPM reflect occupational exposure. The average age at diagnosis ranges from 50 to 70 years [1], with a clear predominance in males, with over 80% of patients being men (M/F ratio of 4/1) [5]. In a data analysis between 1994 and 2008, the mortality rate was estimated at 4.9/1000000, around the age of 70, with a higher mortality rate observed among women (1:2) [8].

The pathophysiological mechanism of mesothelioma is complex and multifactorial. Once inhaled, asbestos fibers migrate to the pleura, where they initiate a process of chronic irritation, followed by repeated cyclical damage and tissue repair. In the pleural space, asbestos fibers are phagocytosed by macrophages, releasing reactive oxygen species that cause DNA damage and lead to abnormal repair processes. Additionally, asbestos fibers can penetrate directly into mesothelial cells, where they interfere with mitosis, induce DNA mutations, and alter chromosomal structure. Exposure of mesothelial cells to asbestos particles triggers the release of inflammatory cytokines such as tumor growth factor-β, platelet-derived growth factor, and vascular endothelial growth factor (VEGF), which contribute to creating an environment conducive to tumor development. Furthermore, asbestos stimulates the phosphorylation of protein kinases, leading to increased expression of proto-oncogenes and promoting abnormal cellular proliferation [9-11].

Clinical manifestations of malignant pleural mesothelioma are polymorphic, nonspecific, and often present late, which frequently delays diagnosis. The clinical picture includes persistent, dull chest pain, dyspnea, cough, and constitutional symptoms such as weight loss, fever, night sweats, and fatigue, which progress as the disease advances. In some cases, digital clubbing, osteoarthropathy, and gynecomastia may be observed. The pain is characteristically non-pleuritic and is often felt in the upper abdominal region or the ipsilateral shoulder due to diaphragmatic involvement [1,5,12].

In our case, the symptoms align with the medical literature: initially presenting with exertional dyspnea, right-sided thoracic pain, and low-grade fever, which progressively evolved to include additional symptoms such as weight loss and asthenia. The lack of specificity of these symptoms, combined with the delayed hospital presentation and the initial labeling of the clinical picture as potentially infectious, contributed to a deviation (fade of a) from a clear diagnostic path. Additionally, a diagnostic delay occurred due to the patient's choice to postpone thoracoscopy with pleural biopsy. Early diagnosis is crucial, but it is a challenging goal given that symptoms typically arise in advanced stages of the disease.

Chest X-rays have limited sensitivity and specificity and are not particularly useful for evaluating pleural effusions. However, they remain the primary diagnostic tool from which suspicion often originates. They can reveal large or moderate pleural effusions and, after drainage, may show pleural thickening with classic features such as mamelonated appearance, pleural plaques, and/or calcifications [1,13,5].

CT scans offer greater diagnostic accuracy, are qualified to identify suggestive features such as nodular pleural thickening, and can capture the involvement of fissures through thickening of the pleural fissures. CT is also useful for assessing extension to the pericardium, diaphragm, mediastinal lymph nodes, and distant metastases [1,14,15]. In the second CT evaluation, the crenulated appearance of the pleura, with focal areas of pleural thickening paravertebrally and posteriorly on the right side, as well as the recurrence of pleural effusion, led to the consideration of malignant mesothelioma.

Other imaging investigations such as PET-CT have moderate sensitivity and specificity and are not routinely recommended for diagnosing MPM. Their utility lies in evaluating metastases, assessing mediastinal lymph node involvement, and differentiating from benign asbestos-related pleural conditions. The total glycolytic volume can also provide prognostic value, and monitor disease response [1,15,16]. During oncological follow-up in the current case, the PET scan from February 2024 provided additional evidence for disease recurrence, subsequently leading to the decision to initiate radiotherapy.

MRI has a limited role in diagnosis but can be beneficial for accurate staging and assessing the extent of disease involvement in the thoracic wall, spinal cord, and mediastinum in patients considered for surgery [12,14].

Histological classification of malignant pleural mesothelioma subdivides it into three subtypes, with the most common being the epithelial subtype (~60%), followed by sarcomatoid (20%) and biphasic or mixed (20%, if composed of at least 10% of both subtypes). Additionally, the histopathological subtype provides prognostic significance and influences the extent of the clinical picture [1,5,17].

According to the literature, epithelial mesothelioma has the best prognosis, with an average survival rate of 13 months, followed by the biphasic subtype, with the sarcomatoid subtype having the poorest prognosis, with an average survival rate of four months [1,12,17,18]. Overall, the average survival rate ranges from four to 12 months from diagnosis, indicating a grim prognosis despite advances in diagnostic and therapeutic methods.

Other unfavorable prognostic factors are thoroughly described, including advanced age, male gender, as well as systemic inflammation markers such as elevated levels of C-reactive protein or lactate dehydrogenase, anemia, thrombocytosis, leukocytosis, or an increased neutrophil/lymphocyte ratio. However, performance status and histological type remain the only consistent and clinically significant prognostic factors [1,19-21].

Research regarding biomarkers serologically dosed or in the pleural fluid has generated particular interest due to their potential diagnostic role or utility in treatment monitoring. In this context, the United States Food and Drug Administration (FDA) has validated mesothelin as a useful marker in malignant pleural mesothelioma, contributing to the assessment of response to chemotherapy and detection of recurrences [22,23]. Other markers, such as osteopontin and fibulin-3, have limited practical utility [24].

Despite medical and technological advances, there are still controversies regarding standard care, with malignant mesothelioma remaining, to date, devoid of remarkable outcomes. The real effectiveness of available therapeutic interventions in prolonging patient survival is questionable, with the neoplasm burdened by a low survival rate. However, information related to therapeutic innovations, particularly in the field of immunotherapy, offers patients hope for better survival rates.

Therapeutic management strategies include surgical interventions, chemotherapy, and radiotherapy, with the primary goal of symptom relief and survival prolongation. Factors contributing to therapeutic decision-making include tumor size, extent of invasion into adjacent structures, disease stage, patient age, and the presence of comorbidities [25].

The role of surgery in the management of MPM remains a topic of debate. The extent of the surgical intervention is determined based on the stage of the disease, the functional and general status of the patient, planned adjuvant therapy (chemotherapy/radiotherapy), and the experience of the surgical team [1,12]. Extensive surgical interventions (e.g., extrapleural pneumonectomy) or limited procedures (e.g., video-assisted thoracoscopic pleurectomy) do not confer survival benefits in mesothelioma. Moreover, the extent of the surgery dictates the degree of postoperative complications [1,11]. Surgical resections accompanied by chemotherapy and radiotherapy have demonstrated improved survival.

## Conclusions

This case report serves as a reminder of the aggressive and recurrent nature of MPM, as well as the critical need for early diagnosis, multidisciplinary treatment, and ongoing research to explore more effective therapies. Guided by a broad multidisciplinary team, the patient has thus surpassed the survival statistics estimated in the medical literature. The diagnosis established in May 2021 had undergone all therapeutic stages, from the challenging surgical procedure with potential major risks to chemotherapy and radiotherapy until June 2024. Prolonged survival is the primary notable aspect of this case, complemented by the maintenance of good performance status up to this point. Moreover, the association of the two episodes of SARS-CoV-2 infection, in mild to moderate form, may be identified as potential triggers that could have altered the trajectory of the neoplastic disease, due to the added immunosuppression and the potential complications of COVID-19 infection.

The case also underscores the urgent need for early detection and innovative treatment strategies in managing MPM, highlighting how delayed diagnosis exacerbates disease progression and restricts therapeutic efficacy. Despite advancements in multimodal treatments including surgery, chemotherapy, and radiotherapy, the aggressive nature and recurrence of MPM reveal limitations in current options. The effectiveness of routine imaging in early recurrence detection is crucial, yet the case illustrates that conventional therapies alone may be insufficient. The continued resistance of MPM to standard treatments signals a critical need for research into targeted therapies and immunotherapy, which could ultimately reshape the survival landscape for MPM patients.
